# Dependence on NIRS Source-Detector Spacing of Cytochrome *c* Oxidase Response to Hypoxia and Hypercapnia in the Adult Brain

**DOI:** 10.1007/978-1-4614-7411-1_47

**Published:** 2013-03-25

**Authors:** Christina Kolyva, Arnab Ghosh, Ilias Tachtsidis, David Highton, Martin Smith, Clare E. Elwell

**Affiliations:** 00471 Department of Medical Physics and Bioengineering, University College London, London, UK; 00472 Neurocritical Care Unit, University College Hospitals, London, UK

**Keywords:** Arterial Oxygen Saturation, Optical Scattering, Adult Head, Frequency Domain Measurement, Discrete Wavelength

## Abstract

Transcranial near-infrared spectroscopy (NIRS) provides an assessment of cerebral oxygen metabolism by monitoring concentration changes in oxidised cytochrome *c* oxidase Δ[oxCCO]. We investigated the response of Δ[oxCCO] to global changes in cerebral oxygen delivery at different source-detector separations in 16 healthy adults. Hypoxaemia was induced by delivery of a hypoxic inspired gas mix and hypercapnia by addition of 6 % CO_2_ to the inspired gases. A hybrid optical spectrometer was used to measure frontal cortex light absorption and scattering at discrete wavelengths and broadband light attenuation at 20, 25, 30 and 35 mm. Without optical scattering changes, a decrease in cerebral oxygen delivery, resulting from the reduction in arterial oxygen saturation during hypoxia, led to a decrease in Δ[oxCCO]. In contrast, Δ[oxCCO] increased when cerebral oxygen delivery increased due to increased cerebral blood flow during hypercapnia. In both cases the magnitude of the Δ[oxCCO] response increased from the detectors proximal (measuring superficial tissue layers) to the detectors distal (measuring deep tissue layers) to the broadband light source. We conclude that the Δ[oxCCO] response to hypoxia and hypercapnia appears to be dependent on penetration depth, possibly reflecting differences between the intra- and extracerebral tissue concentration of cytochrome *c* oxidase.

## Introduction

Transcranial near-infrared spectroscopy (NIRS) provides a measure of cerebral oxygen delivery by monitoring concentration changes in oxygenated (Δ[HbO_2_]) and deoxygenated haemoglobin (Δ[HHb]), non-invasively. Concentration changes in oxidised cytochrome *c* oxidase (Δ[oxCCO]) can also be derived with NIRS. Cytochrome *c* oxidase (CCO) is the terminal electron acceptor in the mitochondrial respiratory chain, and being responsible for over 95 % of oxygen metabolism, it is instrumental in aerobic ATP synthesis and in maintaining mitochondrial function [1]. Since in the short term the total concentration of CCO does not change, changes in the Δ[oxCCO] signal track changes in the CCO redox state, which essentially reflects the balance between cerebral energy supply and demand [2]. Thus, Δ[oxCCO] is an appealing target for bedside monitoring, for the assessment of regional cerebral metabolic status and oxygen utilisation.

Technical complexities are associated with the measurement of Δ[oxCCO] in the adult brain, in the presence of significantly higher concentrations of haemoglobin, most notably the possible interference of changes in optical scattering with the NIRS measurements [3, 4] and the insufficient chromophore separation by the algorithm used to convert optical density into concentration changes [3, 4]. A hybrid optical spectrometer (pHOS) with the capacity for measurements at multiple interoptode distances (and thus at multiple depths) and accompanying algorithm designed to address the above issues have recently been developed by our group [5]. The capacity for multi-distance Δ[oxCCO] recordings would contribute considerably to the interpretation of this measurement, by determining if there is a distance/depth-dependent response of Δ[oxCCO] in the adult head [6–8].

The aim of the present study was to investigate the multi-depth response of Δ[oxCCO] to global changes in cerebral oxygen delivery driven by systemic hypoxia and hypercapnia. We hypothesised that Δ[oxCCO] would show an incremental response with increasing source-detector separation, mirroring potential differences in the extra- and intracranial distribution of this chromophore.

## Methods

### Study Population

A total of 16 adult healthy volunteers were studied (Table 47.1). The studies were approved by the local ethics committee and all subjects provided written informed consent.

**Table 47.1:** Patient demographics and systemic variables

	**Hypoxia**		**Hypercapnia**	
**n**	15		12	
**Age**	30 (22–35)		30 (25–34)	
**Gender**	10 male		8 male	
	**Baseline**	**End challenge**	**Baseline**	**End challenge**
**SpO**_**2**_ *(%)*	97 (93–100)	80 (70–86)^*^	98 (93–100)	97 (93–100)
**EtCO**_**2**_ *(kPa)*	5.5 (3.9–6.4)	5.2 (4.2–6.4)^*^	5.5 (4.5–6.0)	7.8 (6.3–9.2)^*^
**Vmca** *(Δ% from rest)*	0	14.3 (−5.4–53.2)^*^	0	57.4 (21.8–87.0)^*^

Table entries are mean (range)

^*^ *P* < 0.05

### Protocol

Hypoxaemia was induced by delivery of a hypoxic gas mix, using a sequential gas delivery circuit. Following 5 min of air inhalation (‘*baseline*’), nitrogen was added to the inspired gas and titrated to produce a progressive reduction in arterial oxygen saturation (SpO_2_) to 80 %, whilst maintaining constant end-tidal partial pressure of carbon dioxide (EtCO_2_). SpO_2_ was sustained at 80 % for 5 min, before returning the inspired gas to room air. Upon reaching normoxia, 5 min of baseline completed the sequence. Hypercapnia was induced by addition of 6 % CO_2_ to the inspired gas mix, after 5 min of initial baseline. This mix was inhaled for another 5 min, before the inspired CO_2_ fraction was returned to 0. When normocapnia was restored, 5 min of baseline concluded the protocol.

### Instrumentation

The pHOS, described in more detail elsewhere, was used for frontal near-infrared measurements during changes in the composition of the inspired gases [5]. The pHOS combines frequency domain (FD) and broadband (BB) components and can measure light absorption and scattering at discrete wavelengths (690, 750, 790 and 850 nm), together with BB light attenuation in the range 504–1,068 nm. Each pHOS optode incorporates an FD channel (source-detector spacing 30 and 35 mm) and BB channel (source-detector spacing 20, 25, 30 and 35 mm). One optode positioned ipsilateral to other cerebral monitoring was used. Each sampling cycle of the pHOS lasts 3.2 s, and BB and FD measurements are sequential. Systemic recordings included beat-to-beat SpO_2_, continuous measurements of inspired/expired oxygen and CO_2_ partial pressure and middle cerebral artery flow velocity, measured with transcranial Doppler ultrasonography.

### Data Analysis

Data analysis was performed in Matlab (version R2010b, Mathworks). Δ[HbO_2_], Δ[HHb] and Δ[oxCCO] were determined from the 780–900 nm portion of the BB attenuation change data using the UCLn algorithm, which is based on the modified Beer-Lambert law. Differential pathlength factor (DPF) was assumed to be 6.26 [9] and its wavelength dependence was accounted for [10]. Changes in total haemoglobin concentration were defined as Δ[HbT] = Δ[HbO_2_] + Δ[HHb] and in haemoglobin difference as Δ[Hbdiff] = Δ[HbO_2_] − Δ[HHb]. The concentrations were linearly detrended for removal of baseline drift and low-pass filtered with a fifth-order Butterworth filter (cut-off frequency 0.08 Hz). The absorption (μ_a_) and reduced scattering (μ_s_′) coefficients were quantified from the FD measurements.

Inspired and end-tidal gas partial pressures were derived from the positive and negative envelopes of the partial pressure waveforms. Separately for each volunteer, the beginning of the induction to hypoxia/hypercapnia and end of hypoxia/hypercapnia were identified from the O_2_/CO_2_ envelopes, with the period between the two points denoted as ‘challenge’. To enable data averaging across subjects despite potential variation in the timing of their response, the challenges were the split into eight phases; hypoxia was split into four equal phases corresponding to the gradual fall in SpO_2_ and another four corresponding to the plateau of 80 % SpO_2_, whilst hypercapnia was split in eight equal phases. These eight phases represented time intervals ‘2–9’ of each challenge. Time interval ‘1’ corresponded to the baseline immediately prior to the induction, whilst time intervals ‘10–17’ covered the period after the end of the challenge; all these intervals mirrored in duration interval ‘2’. Representative optical and systemic data for each of the 17 time intervals were derived by averaging the last 9*3.2 s worth of data of the corresponding 17 data segments.

### Statistical Analysis

SPSS was used (version 18.0, IBM). Normality was assessed with Q-Q plots. Repeated measures ANOVA tests with Greenhouse-Geisser correction determined whether the group means overall changed significantly between time points 1 and 17. Post hoc tests with Bonferroni corrections established the time points with a statistically significant change compared to point 1. Average data are expressed as mean ± SD and statistical significance was assumed at *P* < 0.05.

## Results

Summary demographic data, separately for the two challenges, are given in Table 47.1. The table also includes group data at baseline and at the end of the challenge (time point 9) for a number of systemic parameters.

**Fig. 47.1:**
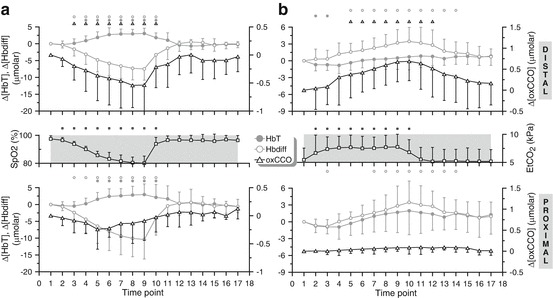
(**a**) Grand averages of the time courses of Δ[HbT], Δ[Hbdiff] and Δ[oxCCO] measured by the detectors distal (*top*) and proximal (*bottom*) to the light source, over the 15 volunteers that participated in the hypoxia challenge. The corresponding arterial oxygen saturation trace (SpO_2_) is also provided for reference. The small symbols on top of each plot indicate statistical significance with respect to point 1 (*P* < 0.05) for the parameters plotted in matching symbols. (**b**) Grand averages of the time courses of Δ[HbT], Δ[Hbdiff] and Δ[oxCCO] measured over the 12 volunteers that participated in the hypercapnia challenge. The corresponding end-tidal CO_2_ trace (EtCO_2_) is also provided for reference. The small symbols on top of each plot indicate statistical significance with respect to point 1 (*P* < 0.05)

Group grand averages of the time courses of Δ[oxCCO], Δ[HbT] and Δ[Hbdiff] as measured during hypoxia by the two extreme detectors are shown in Fig. 47.1a. During hypoxia, the detector distal to the light source recorded a decrease in Δ[oxCCO] (*P* < 0.001), in agreement with previous studies [6], which was accompanied by an increase in Δ[HbT] (*P* < 0.001) and decrease in Δ[Hbdiff] (*P* < 0.001). In terms of directional changes, the findings were qualitatively similar for all detectors, but the magnitude of the Δ[oxCCO] response to hypoxia gradually increased from the proximal to the distal detectors.

Figure 47.1b displays the group time courses of Δ[oxCCO], Δ[HbT] and Δ[Hbdiff] during hypercapnia. The distal detector registered an increase in Δ[oxCCO] (*P* < 0.001), in agreement with previous studies [11], accompanied by an increase in both Δ[HbT] (*P* < 0.05) and Δ[Hbdiff] (P < 0.001). These trends were similar for all detectors, but the magnitude of the Δ[oxCCO] response to hypercapnia gradually increased from the proximal to the distal detectors.

No changes in μ_s_′ (P = NS for all wavelengths) were measured during hypoxia or hypercapnia.

## Discussion and Conclusions

These first multi-depth Δ[oxCCO] measurements during global changes in cerebral oxygen delivery have revealed an increase in the amplitude of the Δ[oxCCO] response with increasing penetration depth. This dependence is most likely mirroring differences in the concentration distribution of CCO in the adult head. It has been suggested that ∆[oxCCO] is a brain-specific signal on account of higher concentrations of CCO present in the brain than the skin, due to higher mitochondrial density [6–8], but evidence for the existence of such a distribution in the adult head has not been provided before, partly because the necessary data were not technologically possible to obtain in humans in vivo.

With measurements from the distal detector therefore being representative of cerebral events, our results support the findings of previously published studies that were conducted with a single source-detector pair and could thus not confirm with certainty that the changes in the head Δ[oxCCO] signal they reported during manipulation of cerebral oxygen delivery were indeed of cerebral origin [6, 11]. In healthy adults, a decrease in cerebral oxygen delivery, induced by a reduction in arterial oxygen saturation during moderate hypoxia, was followed by a 0.24 μmolar decrease (median) in Δ[oxCCO], indicating reduced cellular oxygen availability [6]. We measured a decrease of 0.55 μmolar (median), with the same interoptode spacing as [6] (3.5 cm). Analogously, an increase in oxygen delivery, via increased cerebral blood flow during hypercapnia, was accompanied by a 0.25 ± 0.17 μmolar increase in Δ[oxCCO], indicating that at normoxic normocapnia CCO is not fully oxidised [11]. In agreement with [11], our data show an increase of 0.69 ± 0.46 μmolar at the same interoptode spacing (3.5 cm). Some animal data suggest that CCO is fully oxidised in normoxia [12]. However, our studies were carried out in healthy awake humans rather than anaesthetised animals, which may have a significant impact on the relationship between oxygen supply and demand and CCO oxidation.

The pHOS system has been specifically optimised for monitoring Δ[oxCCO], by combining measurements of light absorption and scattering at discrete wavelengths with multi-distance measurements of BB light attenuation. No change in optical scattering (a potential confounding influence on Δ[oxCCO] measurements) was measured. Moreover, the use of BB light for resolving chromophores over hundreds of wavelengths gave confidence that the algorithm used to convert optical density to concentration changes would provide sufficient chromophore separation.

We conclude that the Δ[oxCCO] response to hypoxia and hypercapnia appears to be dependent upon penetration depth, possibly reflecting differences between the intra- and extracerebral tissue concentration of CCO.
